# Grad-CAM-Based Investigation into Acute-Stage Fluorescein Angiography Images to Predict Long-Term Visual Prognosis of Branch Retinal Vein Occlusion

**DOI:** 10.3390/jcm13175271

**Published:** 2024-09-05

**Authors:** Michiyuki Saito, Mizuho Mitamura, Mayuko Kimura, Yuki Ito, Hiroaki Endo, Satoshi Katsuta, Manabu Kase, Susumu Ishida

**Affiliations:** 1 Department of Ophthalmology, Faculty of Medicine and Graduate School of Medicine, Hokkaido University, N-15, W-7, Kita-ku, Sapporo 060-8638, Japan; 2 Department of Ophthalmology, Teine Keijinkai Hospital, Sapporo 006-0811, Japan

**Keywords:** visual prognosis, branch retinal vein occlusion, fluorescein angiography, deep learning, explainable artificial intelligence, gradient-weighted class activation mapping (Grad-CAM), ResNet

## Abstract

**Background/Objectives**: The purpose of this study was to analyze relevant areas in acute-stage fluorescein angiography (FA) images, predicting the long-term visual prognosis of branch retinal vein occlusion (BRVO) based on gradient-weighted class activation mapping (Grad-CAM). **Methods**: This retrospective observational study included 136 eyes with BRVO that were followed up for more than a year post-FA. Cropped grayscale images centered on the fovea (200 × 200 pixels) were manually pre-processed from early-phase FA at the acute phase. Pairs of the cropped FA images and the best-corrected visual acuity (BCVA) in remission at least one year post-FA were used to train a 38-layer ResNet with five-fold cross-validation. Correlations between the ResNet-predicted and true (actually measured) logMAR BCVAs in remission, and between the foveal avascular zone (FAZ) area measured by ImageJ (version 1.52r) from FA images and true logMAR BCVA in remission were evaluated. The heat maps generated by Grad-CAM were evaluated to determine which areas were consumed as computational resources for BCVA prediction. **Results**: The correlation coefficient between the predicted and true logMAR BCVAs in remission was 0.47, and that between the acute-stage FAZ area and true logMAR BCVA in remission was 0.42 (*p* < 0.0001 for both). The Grad-CAM-generated heat maps showed that retinal vessels adjacent to the FAZ and the FAZ per se had high selectivity (95.7% and 62.2%, respectively). **Conclusions**: The Grad-CAM-based analysis demonstrated FAZ-neighboring vessels as the most relevant predictor for the long-term visual prognosis of BRVO.

## 1. Introduction

Branch retinal vein occlusion (BRVO), the obstruction of the retinal venous branches, can result in serious visual impairment. Macular edema (ME) and serous retinal detachment (SRD) are known factors which can influence visual acuity in BRVO; however, there have been cases of poor improvement in visual acuity despite the resolution of ME and SRD with treatment [1]. Factors influencing the visual acuity of BRVO in remission include the enlargement of the foveal avascular zone (FAZ) [2,3] and vessel density around the central fovea [4,5,6,7]. Macular ischemia at the acute stage may be associated with the long-term visual prognosis of BRVO; however, this relationship remains unclear.

With the ongoing development of artificial intelligence (AI)-based image analysis in recent years, a wide range of research using deep learning has been published in the field of ophthalmology, from big [8,9] to small data [10,11,12,13,14]. The problem with AI research, known as the “black box” problem, is that it is unclear what the AI is basing its decisions on among the input data. Therefore, there is a need to achieve explainable AI in the medical field, with which to explain various models and their results in a manner that humans can understand. An explanatory visualization method, known as gradient-weighted class activation mapping (Grad-CAM), is used to generate heat maps that can provide the basis for intuitive decision-making through the use of various models [15]. It is expected that the basis for AI decision-making will yield new scientific knowledge by providing unexpected perspectives that humans have not previously focused on.

This study aimed to use deep learning to estimate the long-term visual prognosis of BRVO with acute-stage fluorescein angiography (FA) images and to analyze the vision-predicting areas based on Grad-CAM, which visualizes the regions that provide the basis for AI decision-making.

## 2. Materials and Methods

### 2.1. Study Subjects

The protocol for this retrospective observational study adhered to the principles of the Declaration of Helsinki and was approved by the institutional review boards of Teine Keijinkai Hospital (IRB no.1-024078-00) and Hokkaido University Hospital (IRB no.C-T2024-0301) on an opt-out basis, in which patients were given the opportunity to refuse to participate via the website, due to the non-invasive retrospective nature of the study. The study included 136 eyes from 136 Japanese patients (age, 72.3 ± 10.5 years; male-to-female ratio, 9:5) who were diagnosed with treatment-naïve BRVO at the Departments of Ophthalmology at Teine Keijinkai and Hokkaido University Hospitals between December 2013 and July 2018, and underwent at least a year of post-FA follow-up.

All patients underwent ophthalmic examinations, including logMAR best-corrected visual acuity (BCVA), intraocular pressure, slit-lamp microscopy, funduscopy, and FA (Spectralis HRA; Heidelberg Engineering, Heidelberg, Germany and F-10; NIDEK, Gamagori, Japan) from May 2019 to November 2020. Except for FA, the ophthalmological examinations were performed at every regular visit for at least a year after the initial FA examination. 

The exclusion criteria were as follows: (1) patients with other macular complications, such as epiretinal membrane; (2) patients with opaque media to the extent that it can interfere with FA imaging; and (3) patients allergic to contrast media who could not undergo FA.

### 2.2. Deep Learning-Based Prediction of logMAR BCVA in Remission from FA Images 

**Figure 1:**
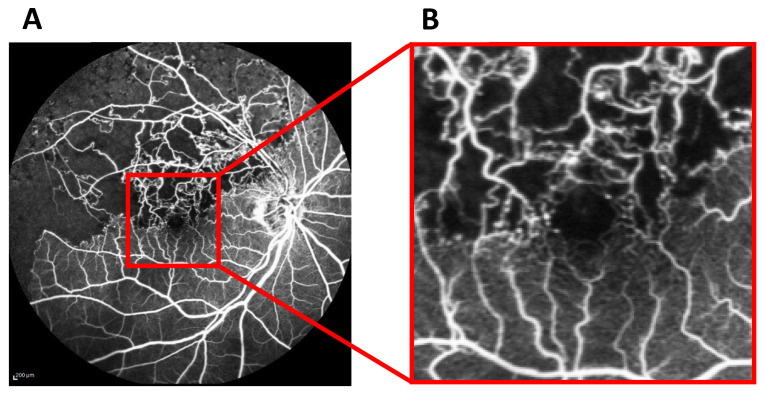
Prediction of logMAR best-corrected visual acuity (BCVA) in remission of branch retinal vein occlusion (BRVO) from fluorescein angiography (FA) images using deep learning. (**A**) An RGB-format FA image with 768 × 868 pixels obtained at the acute stage of BRVO. (**B**) A 200 × 200-pixel image centered on the central fovea of the early-phase FA image was cropped and converted to grayscale.

In this study, the best logMAR BCVA during the period of ME and/or SRD resolution at least one year post-FA was defined as logMAR BCVA in remission. Both the predicted and true logMAR BCVA values were linearly normalized to range between 0 and 1. The early-phase FA grayscale images obtained at the acute stage were cropped within 2 disc diameters (Figure 1A, red square) and resized to 200 × 200-pixel images centered on the central fovea (Figure 1B). Pairs of the preprocessed 200 × 200-pixel FA images and logMAR BCVA in remission were then used for training and validating the neural network.

**Figure 2:**
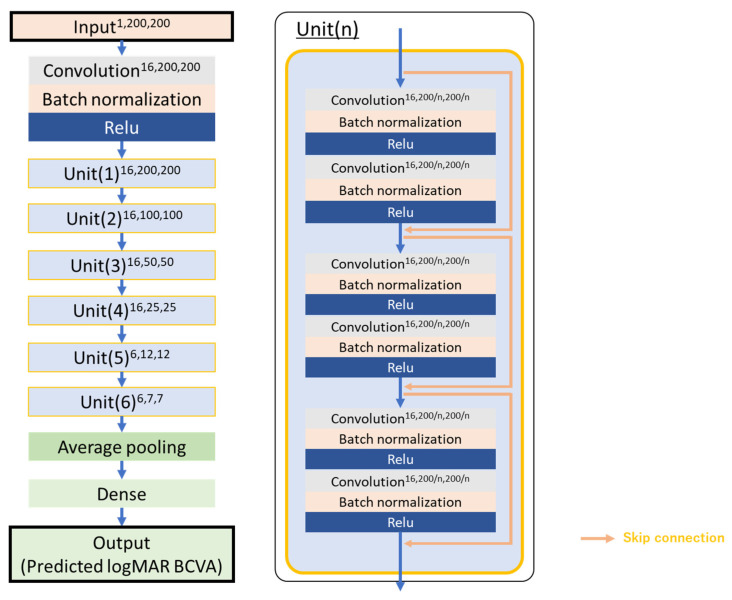
ResNet for predicting logMAR BCVA in remission from acute-stage FA images. The ResNet is a neural network with 38 layers that uses 37 convolution layers and 17 residual networks.

We implemented the neural network on NNabla version 1.33.1 (Sony Corporation, Tokyo, Japan), using a 38-layer ResNet [16] with 37 convolution layers, and 17 residual networks were used to maintain the layer depth to prevent gradient loss and increase the number of layers (Figure 2). Five-fold cross-validation was used to train and validate the neural network.

### 2.3. Visualization of Relevant Areas for BCVA Prediction Using Grad-CAM Heat Maps Merged with Acute-Stage FA Images

**Figure 3:**
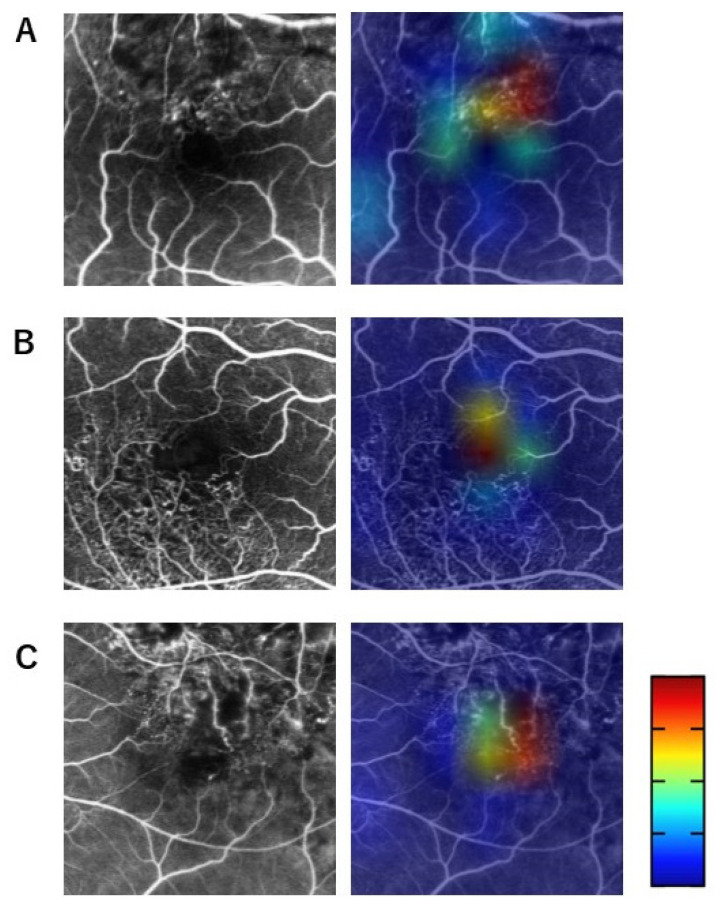
Representative cases of FA images (left) and gradient-weighted class activation mapping (Grad-CAM) (right). The responses to the questionnaire by the two ophthalmologists were consistent with the heat map locations of abnormal retinal vessels adjacent to FAZ (**A**), FAZ and normal retinal vessels adjacent to FAZ (**B**), and FAZ enlargement (**C**).

We used Grad-CAM with heat maps to visualize regions in the FA images that the neural network focused on, according to previous reports [15,17]. As shown in Figure 3, the warmer the color is, the more the neural network focuses on. Using the 136 Grad-CAM heat maps, two ophthalmologists (M.S. and M.K.) independently completed a questionnaire based on heat maps to determine which areas in the acute-stage FA images were identified by the neural network as influencing the predicted logMAR BCVA in remission. The questionnaire items included (multiple answers were allowed): (a) FAZ, (b) FAZ enlargement, (c) normal retinal vessels adjacent to FAZ, (d) abnormal retinal vessels adjacent to FAZ, (e) normal retinal vessels away from FAZ, (f) abnormal retinal vessels away from FAZ, (g) NPAs adjacent to FAZ, (h) NPAs away from FAZ, and (i) unknown.

### 2.4. Endpoints and Statistical Analyses

The primary endpoint of this study was the correlation between the ResNet-predicted and true logMAR BCVAs in remission. For comparison, the acute-stage FAZ area in the FA images was manually measured using ImageJ (National Institutes of Health), and the correlation between the acute-stage FAZ area and true logMAR BCVA in remission was evaluated. Questionnaires on the heat maps generated by Grad-CAM were tabulated, and the percentage of areas AI focused on to predict visual acuity was summarized as descriptive statistics.

Statistical analyses were performed using the R package (version 3.6.1, R Foundation for Statistical Computing, Vienna, Austria). Pearson’s product-moment correlation was used to analyze the correlations between the predicted and true logMAR BCVAs in remission and between the FAZ area and true logMAR BCVA. Statistical significance was set at *p* < 0.05.

## 3. Results

### 3.1. Correlations between the Predicted and True logMAR BCVAs in Remission, and between the Acute-Stage FAZ Area and True logMAR BCVA in Remission

**Figure 4:**
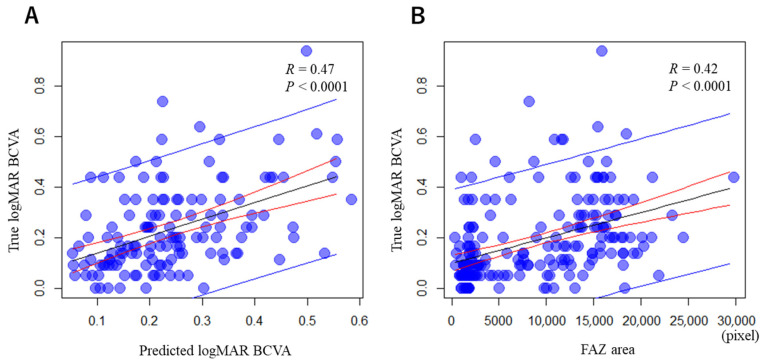
Correlations between the predicted and true logMAR BCVAs in remission (**A**) and between FAZ area and true logMAR BCVA (**B**). There were statistically significant correlations between the ResNet-predicted logMAR BCVA in remission and the true logMAR BCVA in remission (R = 0.47) (**A**) and between the acute-stage FAZ area and the true logMAR BCVA in remission (R = 0.42) (**B**). Compared to FAZ area, predicted logMAR BCVA in remission had a higher correlation with true logMAR BCVA in remission. *p* < 0.0001, Pearson’s product-moment correlation. Blue oblique lines indicate prediction intervals, while red oblique lines mean confident intervals.

As shown in Figure 4, the ResNet-predicted logMAR BCVA in remission based on the acute-stage FA images positively correlated with the true logMAR BCVA in remission (R = 0.47; *p* < 0.0001) (Figure 4A). The acute-stage FAZ area also positively correlated with the true logMAR BCVA in remission (R = 0.42; *p* < 0.0001) (Figure 4B). Compared to the FAZ area, the predicted logMAR BCVA in remission had a higher correlation with the true logMAR BCVA in remission.

### 3.2. Questionnaire on Grad-CAM Heat Maps

As shown in Table 1, the kappa coefficients, which indicated the agreement rate between the two examiners, were ≥75.2%, indicating high agreements. Among the questionnaire items, (a) FAZ, (c) normal, and (d) abnormal retinal vessels adjacent to the FAZ were common, with selectivities of 62.2, 60.0, and 57.2%, respectively. The questionnaire items (c) normal + (d) abnormal retinal vessels adjacent to the FAZ were particularly high at a selectivity of 95.7% (multiple answers allowed), suggesting that FAZ-neighboring retinal vessels were important for analysis in the prediction of visual acuity in remission. The questionnaire items (b) FAZ enlargement, (g) NPA adjacent to, and (h) NPA away from the FAZ were low, with selectivities of 19.1, 12.9, and 9.1%, respectively, suggesting that the FAZ enlargement and NPA were poorly involved in the AI’s prediction of visual acuity.

Figure 3 shows representative Grad-CAM heat maps merged with the early-phase FA images at the acute stage of BRVO. The responses to the questionnaire by the two ophthalmologists were consistent with the heat map locations of (d) abnormal retinal vessels adjacent to the FAZ in Figure 3A, (a) FAZ and (c) normal retinal vessels adjacent to the FAZ in Figure 3B, and (b) FAZ enlargement in Figure 3C.

## 4. Discussion

This study revealed the following important findings on the visual prognosis of BRVO: (1) the deep-learning-based long-term logMAR BCVA in remission predicted from acute-stage FA images showed a moderate correlation with the true logMAR BCVA in remission and had a stronger correlation with the true logMAR BCVA in remission than the acute-stage FAZ area did, and (2) the neural network focused more on retinal vessels adjacent to the FAZ than on the FAZ per se for logMAR BCVA prediction. 

The scientific value of this study is that AI predicted long-term visual prognosis of BRVO from conventional acute-stage FA images and showed favorable predictive accuracy. The correlation between the FAZ-neighboring vascular architecture and simultaneous visual acuity in cases of BRVO has been reported using optical coherence tomography angiography (OCTA), resulting in a vessel area and density correlated more strongly with simultaneous visual acuity than the FAZ area [2,4,5]. However, there has been no report predicting long-term visual prognosis from retinal-vasculature imaging, including the acute-stage FA or OCTA findings. This is the first study to predict long-term visual outcomes in BRVO from acute-stage FA images. A moderate positive correlation was found between true and AI-predicted long-term visual prognosis in this study. These data indicate that the long-term visual prognosis is determined to some extent by the acute-phase condition in BRVO, while it simultaneously suggests that the treatment and other factors after the onset may affect the visual prognosis. 

In the present study, the Grad-CAM-generated heat maps showed the FAZ-neighboring vessels and the FAZ per se as the relevant predictors for long-term visual prognosis, rather than FAZ enlargement and NPA. Although FA is inferior to OCTA in depicting vascular architecture per se, it preserves various information which lacks in OCTA images, including fluorescent leakage and vessels with very slow flow, such as part of microaneurysms in diabetic retinopathy as well as BRVO and polypoidal lesions in polypoidal choroidal vasculopathy. OCTA is a digitally processed image, in which the moving signals from red blood cells are reconstructed in multiple images of the same area. The signal is thus extracted and amplified, and only the vascular structures are depicted with unnecessary information omitted. Therefore, the quality of the information is fundamentally different between FA and OCTA images. FA is superior to OCTA in that it retains unprocessed information in the image. Studies of the same technique using OCTA may theoretically give completely different decision criteria and are within the scope of our future projects to contrast these two modalities. 

The Grad-CAM-based analysis of FA features is likely to serve as a clinically significant platform for better understanding the underlying pathologies that cause poor visual outcomes of BRVO. Since FAZ-neighboring retinal vessels were shown as the most relevant for BCVA prediction, the next issue would reasonably be to investigate which characteristics of these vessels are associated with visual prognosis, including vessel tortuosity, dilation, brightness, formation of microaneurysms, and degree of fluorescent leakage on FA. In this sense, Grad-CAM heat mapping may help identify and point out future research subjects appropriately and efficiently. 

This study had some limitations. First, although the collection of FA images from patients with treatment-naïve BRVO is a strength, this was a retrospective observational study of small sample size, which may have biased the study population, and the results may not be generalizable to a wide range of patients with different demographic and clinical characteristics. Therefore, future prospective large cohorts together with external validation using independent datasets are warranted to further strengthen our results in predicting the logMAR BCVA of patients with BRVO in remission. Second, although Grad-CAM is a useful visualization tool that teaches us the AI-derived relevant areas, heat maps do not explain in detail how particular sites contribute to the prediction, which renders the interpretation of data elusive, but will potentially expand future research. Third, FA images were used for deep learning in this study, but a multimodal strategy with the addition of other imaging modalities and clinical information could have provided more accurate predictive visual acuity.

In conclusion, the FA image-based deep learning successfully predicted logMAR BCVA in remission, which had a stronger correlation with the true logMAR BCVA in remission than the acute-stage FAZ area did. The neural network focused mainly on FAZ-neighboring retinal vessels to estimate the long-term visual prognosis of BRVO. Our deep learning-based prediction of the visual prognosis may be useful in understanding the vision-threatening pathogenesis of BRVO, implicating the validity of its treatment strategy.

## Figures and Tables

**Table 1:** Questionnaire on Grad-CAM heat maps.

Questionnaire Item	Selectivity	Kappa Coefficient
(a) Foveal avascular zone (FAZ)	62.2%	89.8%
(b) FAZ enlargement	19.1%	81.5%
(c) Normal retinal vessels adjacent to FAZ	60.0%	75.2%
(d) Abnormal retinal vessels adjacent to FAZ	57.2%	85.4%
(e) Normal retinal vessels away from FAZ	39.5%	89.0%
(f) Abnormal retinal vessels away from FAZ	24.2%	80.4%
(g) Non-perfusion area (NPA) adjacent to FAZ	12.9%	78.8%
(h) NPA away from FAZ	9.1%	88.4%
(i) Unknown	22.0%	77.7%

## Data Availability

The data that support the findings of this study are available on request from the corresponding author, M.M., and M.S. The data are not publicly available due to their containing information that could compromise the privacy of research participants.
